# The Paradoxical Role of Circulating Ketone Bodies in Glycemic Control of Individuals with Type 2 Diabetes: High Risk, High Reward?

**DOI:** 10.3390/biom12091318

**Published:** 2022-09-18

**Authors:** Amarens van der Vaart, Martine G. E. Knol, Martin H. de Borst, Stephan J. L. Bakker, Margery A. Connelly, Erwin Garcia, Henk J. G. Bilo, Peter R. van Dijk, Robin P. F. Dullaart

**Affiliations:** 1 Department of Internal Medicine, Division of Nephrology, University Medical Center Groningen, University of Groningen, P.O. Box 30.001, 9700 RB Groningen, The Netherlands; 2 Department of Internal Medicine, Division of Endocrinology, University Medical Center Groningen, University of Groningen, P.O. Box 30.001, 9700 RB Groningen, The Netherlands; 3 Laboratory Corporation of America® Holdings (Labcorp), Morrisville, NC 27560, USA; 4 Diabetes Research Center, 8025 AB Zwolle, The Netherlands

**Keywords:** ketone bodies, type 2 diabetes, glycemic control

## Abstract

Introduction: Fasting plasma ketone bodies (KB) are elevated in individuals with type 2 diabetes (T2D) and could affect glycemic control and disease progression. Prolonged KB exposure may result in adaptive beneficial responses, counteracting glycemic dysregulation. In the current proof-of-concept study in adults with T2D, we hypothesized that fasting plasma KB are cross-sectionally associated with poorer glycemic control but prospectively with better glycemic control. Materials and Methods: Fasting plasma KB were measured via nuclear magnetic resonance spectroscopy in patients with T2D treated in primary care (Zodiac cohort; The Netherlands). We analyzed the associations between KB and HbA1c at baseline using linear regression analyses and HbA1c changes over time using linear mixed models. We adjusted for potential confounders, including risk factors for poor glycemic control. Individuals with T2D participating in the general population-based PREVEND study were used as a replication cohort. Results: We included 271 individuals with T2D with a total of 859 HbA1c measurements during a follow-up period of 3.0 (2.0–3.2) years. At baseline, the total amount of fasting plasma KB was independently and positively associated with HbA1c levels (regression coefficient in the fully adjusted analysis = 0.31; 95% CI 0.06–0.57, per doubling of KB; *p* = 0.02). In contrast, in the longitudinal analyses, fasting plasma KB were associated with a yearly HbA1c (%) decrease of −0.10 (95% CI −0.19 to −0.00 per doubling baseline KB; *p* = 0.05). Results were replicated in 387 individuals with T2D from a general population cohort with a total of 1115 glucose measurements during a follow-up period of 7.5 (7.2–8.0) years. A yearly decrease in fasting plasma glucose (mmol/L) of 0.09 was found per doubling of baseline KB. Conclusions: This study is the first to suggest a paradoxical role of circulating KB on glycemic control in T2D: elevated KB are associated with cross-sectionally poorer glycemic control but longitudinally with better long-term glycemic control.

## 1. Introduction

Ketone bodies (KB), including β-hydroxybutyrate (β-OB), acetoacetate, and acetone, serve as an alternative energy source during periods of glucose deprivation and protect against cellular starvation [1]. They are mainly produced as a result of fatty acid oxidation in the liver when there is an accumulation of acetyl-CoA [2]. Upon inability to enter the citric acid cycle, acetyl-CoA is converted to acetoacetate, of which the majority is (reversibly) reduced to β-OHB, and a small part is decarboxylated to acetone. Subsequently, circulating KB are transported to peripheral tissues for metabolism [3]. In healthy individuals, blood KB rise during fasting and prolonged exercise. However, in individuals with type 2 diabetes (T2D), fasting plasma KB levels are pathologically elevated [4] as a result of increased KB production and reduced KB clearance consequent to (relative) insulin deficiency [5]. In clinical studies, elevated KB are found to predict new-onset T2D and mortality in the general population [6,7].

Interestingly, although KB initially induce oxidative stress, as well as enhancing inflammatory responses and cell damage [8,9], in the long term, KB appear to have a paradoxical beneficial effect [10,11]. Animal studies have shown that the adverse short-term effects of KB can be counteracted by favorable long-term adaptive responses [12,13]. For example, prolonged KB exposure in vitro may protect against KB-induced oxidative damage [14,15,16]. In line with this, several clinical studies confirm the long-term beneficial effects of KB in several disease states, such as diabetic nephropathy and heart failure [17,18].

Notably, the putative bidirectional role of plasma KB as a marker of disturbed glycemic control in T2D and the subsequently presumed compensatory responses make it challenging to distinguish such opposing effects in clinical studies. Therefore, in this proof-of-concept observational study, we aimed to test the hypothesis that fasting plasma KB may serve as a biomarker for poor glycemic control in T2D but are also associated with improved metabolic control in the long term.

## 2. Materials and Methods

### 2.1. Study Design and Participants

The Zwolle Outpatient Diabetes project Integrating Available Care (ZODIAC) study is a Dutch cohort of individuals with T2D, which has been described elsewhere [19]. At the start of this prospective cohort study in 1998, 1143 individuals were enrolled. After excluding all individuals with missing plasma KB and HbA1c at baseline, 605 individuals remained. After the exclusion of individuals with missing follow-up HbA1c, we included 271 individuals in the current study. Eighteen percent of T2D treatment data was missing. The remainder of the variables required for statistical modeling were complete.

The main findings of the current proof-of-concept study were replicated in a second independent cohort, the Prevention of Renal and Vascular End-stage Disease (PREVEND) study, which has been described elsewhere [20]. We included 387 individuals with T2D at baseline, fasting plasma KB at baseline, and the availability of longitudinal fasting glucose data.

Approval for the protocol and informed consent procedure for the ZODIAC study was given by the medical ethics committee of the Isala Clinics, Zwolle, the Netherlands (METC reference numbers 03.0316 and 07.0335). Approval of the PREVEND cohort study was given by the medical ethics committee of the University Medical Centre Groningen, the Netherlands (approval number: MEC96/01/022). All procedures were conducted according to the Declaration of Helsinki.

### 2.2. Data Collection

Blood pressure was measured two times with a Welch Allyn sphygmomanometer (Skaneateles Falls, NY, USA) after at least 5 min of rest (measured in a supine position). Body mass index (BMI) was calculated by dividing weight in kilograms by height in meters squared. Height and weight were measured while standing and without shoes. T2D treatment with respect to glucose control was classified according to the traditional treatment steps in nationwide guidelines and consisted of lifestyle interventions, oral blood-glucose-lowering drugs (OBGLD) (mainly metformin and sulfonylurea, but not dipeptidyl peptidase-4 (DPP4) inhibitors, glucagon-like peptide 1 (GLP1) agonists, and sodium–glucose cotransporter 2 (SGLT2) inhibitors) and insulin (both short-acting and prolonged-acting human insulin preparations). Lifestyle interventions include the advice not to smoke, getting enough physical exercise, weight loss if BMI exceeds 25, and dietician advice in case of insufficient weight loss.

### 2.3. Laboratory Methods

Venous blood samples were drawn after an overnight fast and 15 min of rest prior to sample collection. All blood samples were collected at once between 8:00 and 10:00 in the morning. Fasting plasma KB were measured in Ethylenediaminetetraacetic acid (EDTA)—anticoagulated plasma samples using the Vantera^®^ Clinical Analyzer— and a nuclear magnetic resonance (NMR) spectrometer (Labcorp, Morrisville, NC, USA) equipped with a 400 MHz (9.4 T) magnet, as previously described. KB quantified by NMR showed highly similar results compared to mass spectrometry (R^2^ = 0.996, 0.994, and 0.994 for β-OHB, acetoacetate, and acetone, respectively). Coefficients of variation for intra- and inter-assay precision ranged from 1.3% to 9.3%, 3.1% to 7.7%, and 3.8% to 9.1%, for β-OHB, acetoacetate, and acetone, respectively. More information regarding the KB measurements is described in detail elsewhere [4]. Lipids and HbA1c were measured by routine laboratory methods as described [21]. Plasma glucose was measured by dry chemistry (Eastman Kodak, Rochester, NY, USA).

### 2.4. Statistical Analyses

All statistical analyses were performed with R version 3.4.2 (Vienna, Austria). Normality was tested with histograms and probability plots. Total KB, β-OHB, acetoacetate, and acetone were all log-base 2 transformed to yield an approximately normal distribution and to allow for data interpretation per doubling of total and individual KB. Outliers in KB and HbA1c levels were removed according to Z-scores (>2 standard deviations). Continuous variables are expressed in terms of mean ± SD or median (interquartile range). Two-sided *p*-values < 0.05 were considered statistically significant.

For the cross-sectional analyses, linear regression analyses were performed to study the association between KB and HbA1c at baseline. Age, gender, BMI, T2D therapy, systolic blood pressure, smoking, T2D duration, C-reactive protein, and eGFR were added stepwise as potential confounders. The normality of residuals was evaluated by the inspection of Q-Q plots to ensure that the assumptions for linear regression analyses were validated.

Annual changes in HbA1c were assessed using subject-specific slope estimates of a linear mixed model for KB with fixed and random effects for intercept and time [22]. An unstructured covariance structure was used. Time was treated as the main fixed factor in all models. Age, gender, BMI, T2D therapy, systolic blood pressure, smoking, T2D duration, C-reactive protein, and eGFR were added stepwise as potential confounders, including their interaction with time. Furthermore, potential effect modification by T2D therapy was tested, containing both the main effect and its cross-product term. To validate the assumptions of linear mixed modeling, the normality of residuals was evaluated by the inspection of Q-Q plots.

## 3. Results

### 3.1. Baseline Characteristics of ZODIAC 

A total of 271 individuals with T2D were included in the current study. Baseline characteristics are presented in Table 1. Age at the start of the study was 65 ± 10 years, 53% of the study participants were female, and average HbA1c levels at baseline were 7.2 ± 1.2 % (55 ± 13 mmol/mol). Total fasting plasma KB were 182 (147–232) μmol/L, β-OHB was 120 (93–161) μmol/L, acetoacetate was 41 (32–56) μmol/L, and acetone was 18 (12–25) μmol/L.

### 3.2. Cross-Sectional Analyses

The cross-sectional associations between KB and HbA1c were determined in multivariable regression analyses, adjusted for age, gender, BMI, systolic blood pressure, smoking, total cholesterol, HDL cholesterol, eGFR, C-reactive protein, and T2D duration. The results are presented in Table 2. The final model revealed that the total fasting plasma KB concentration was independently and positively associated with HbA1c levels at baseline (fully adjusted estimated unstandardized regression coefficient 0.31 (95% CI 0.06 to 0.57, *p* = 0.01, adjusted R^2^ for KB = 0.02). A similar association was found for β-OHB (fully adjusted unstandardized regression coefficient 0.28 (95% CI 0.08 to 0.47, *p* = 0.01). These results corresponded to a higher average HbA1c (%) of 0.31 and 0.28 for each doubling in plasma total of KB and β-OHB, respectively. No significant associations were found between acetoacetate, acetone, and HbA1c.

### 3.3. Longitudinal Analyses

Subsequently, we performed longitudinal analyses using linear mixed models to assess the association between baseline fasting plasma KB and the change in HbA1c over time. During a follow-up period of 3.0 (2.0–3.2) years, a total of 859 HbA1c measurements were performed, i.e., 4 (3–4) measurements per participant. In all participants combined, HbA1c (%) did not materially change during follow-up (+0.04, *p* = 0.67). In the group with the lowest tertile of plasma KB at baseline, HbA1c increased during follow-up (*p* = 0.04), but it did not significantly change in the other groups (Supplemental Figure S1). As shown in Table 3, higher values of total fasting plasma KB, as well as β-OHB, were associated with a future decrease in HbA1c levels in linear mixed model analyses. These associations remained independent of adjustment for relevant covariates. The final model showed that doubling of fasting plasma KB is associated with a yearly HbA1c (%) decrease of −0.10 (95% CI −0.19 to −0.00, *p* = 0.05, marginal R^2^ for KB = 0.02). The same association was found for β-OHB (fully adjusted estimated regression coefficient = −0.08; 95% CI −0.16 to −0.01, *p* = 0.03). Interestingly, after additional adjustment for T2D therapy in Model 2, the association was close to significance (*p* < 0.10). There was no significant association between acetoacetate and acetone with HbA1c change over time. To study the longitudinal associations of KB and HbA1c according to T2D therapy, stratified analyses by treatment were performed (Supplemental Table S1; missing treatment data in 51 participants). In the largest therapy group using oral blood-glucose-lowering drugs alone, all associations persisted, even in the stratified analysis. In the other therapy groups, the association of KB with HbA1c at follow-up was not significant. Nonetheless, there was an interaction between insulin use and KB impacting HbA1c change over time (p-interaction between insulin use and other treatments, *p* = 0.01 in crude and *p* = 0.02 in the fully adjusted analyses).

### 3.4. Replication Cohort

Finally, we repeated all analyses in an independent replication cohort of 381 individuals with T2D with fasting glucose levels as a marker of glycemic control. The baseline KB were associated with higher fasting plasma glucose levels (fully adjusted estimated regression coefficient = 0.84; 95% CI 0.45 to 1.24, *p* < 0.001, adjusted R^2^ for KB = 0.05). These findings are consistent with the findings for HbA1c in the ZODIAC cohort. Furthermore, we assessed the change in fasting glucose levels using a total of 1115 fasting glucose measurements during a follow-up period of 7.5 (7.2–8.0) years. In the final model, the baseline KB were associated with a yearly decrease in fasting glucose levels (mmol/L) of −0.09 (95% CI −0.15 to −0.02 per doubling baseline KB; *p* = 0.01, marginal R^2^ for KB = 0.03) (Supplementary Table S2).

## 4. Conclusions

In this observational study among individuals with T2D, we investigated the bidirectional role of KB in glycemic control. We found that KB levels were cross-sectionally associated with higher HbA1c levels, but longitudinally with lower HbA1c levels. The beneficial effects of fasting KB with glycemic control were replicated in a population-based cohort using yearly changes in fasting plasma glucose as the outcome. KB are often put in a bad light in the context of diabetes, mostly due to diabetic ketoacidosis. By contrast, the beneficial effects of KB in T2D have barely been discussed. This study provides evidence that increased KB synthesis in T2D could initiate a protective response that favors glycemic control in the long term. To our knowledge, our report is the first to describe such paradoxical effects of KB on glycemic control. We interpret our findings to indicate that plasma KB may serve as relevant biomarkers for more moderate metabolic control but could also be indicative of protective mechanisms against further long-term glycemic dysregulation.

There are some previous studies that have shown the cross-sectional and longitudinal associations of KB separately. In individuals with T2D, KB levels are pathologically elevated, and high plasma KB are predictive of incident T2D in initially non-diabetic individuals [6,23,24]. Additionally, other studies revealed long-term beneficial effects of KB, e.g., improved glycemic control and reduced inflammation [15,16,25]. However, there are no earlier studies investigating the future improvements in metabolic control conceivably as a result of elevated KB levels in individuals with T2D, except in the context of ketogenic diets, which we will discuss further below.

Because of the observational design of the current study, we cannot draw conclusions about causality. However, it seems plausible to hypothesize about potential mechanisms underlying the observed associations. Although KB initially triggered the formation of reactive oxygen species (ROS) and pro-inflammatory mediators, resulting in oxidative stress and cellular damage, this response is followed by a stronger adaptive defense response that eventually results in beneficial effects. The upregulation of danger-responsive genes and mediators, such as Nrf2, but also histone deacetylases of the sirtuin (SIRT) family and AMP-activated kinases, in response to ketonemia, resolves oxidative stress via anti-inflammatory activities and due to improved cellular metabolism [10,13,26].

The rising popularity of ketogenic diets supports the supposed beneficial effects of KB. Several studies showed that ketogenic diets improved glycemic control in those with T2D and protected them against diabetic complications [27,28]. In addition to ketogenic diets, sodium-glucose cotransporter-2 (SGLT-2) inhibitors persistently increase circulating KB in the general population and, to a larger extent, in populations with T2D [29,30]. SGLT-2 inhibitors have emerged as an effective therapy for improving metabolic control and other health outcomes in T2D. Studies report that several beneficial effects of SGLT-2 inhibitors are mediated by induced endogenous KB production; for example, in diabetic nephropathy and cardiac heart disease [31,32]. However, there were no participants using SGLT-2 inhibitors or a ketogenic diet in the present study to further investigate this matter.

Another potential area of interest is the stratified analysis of the longitudinal associations within the different T2D therapy groups, where the associations between KB and change in HbA1c over time would appear to reverse in the group using insulin. This finding may be the result of the small population size, limiting the statistical power to properly address these effects, which also supports why the inverse association did not reach statistical significance. In addition, we can speculate about a potential interaction between insulin therapy and KB metabolism. Previous studies described that hyperinsulinemia stimulates β-OHB consumption and increases KB disposal [33]. Because of this interaction, intermittent hyperinsulinemia might influence the presumed adaptive response to long-term hyperketonemia. Another possible explanation is that the effect of KB as a biomarker of progressive disease outweighs the long-term protective effect in the moderately controlled T2D group compared to the group using diet or OBGLD as therapy.

A major strength of this study is the well-characterized cohort with data regarding T2D status, clinically relevant outcomes, and the availability of repeated glucose and HbA1c measurements over time. Another strength of the current study is that we were able to replicate the main results in another cohort, with additional adjustments for covariates similar to the main cohort. Additionally, due to the use of a replication cohort, we could prove our findings are consistent for changes in HbA1c levels as well as in fasting glucose levels. Furthermore, in the replication cohort, we could also adjust for fasting insulin at baseline and time-updated covariates, including T2D therapy. At the same time, certain limitations should be mentioned. First, in the ZODIAC cohort, almost half of the individuals were excluded because there were no repeated HbA1c data, most likely because patients were referred to secondary care or died, potentially leading to selection bias and incomplete data on T2D (drug) therapy. Second, we only had data about T2D therapy at baseline in the ZODIAC cohort and do not know whether this changed during follow-up, but we were able to take this into account in the replication cohort. Finally, plasma-free fatty acids were not measured. Therefore, we can only speculate that increased lipolysis from adipose tissue has led to a free fatty acids influx in the liver and resulted in increased β-oxidation and ketonemia.

In conclusion, this study suggests a paradoxical role of circulating KB on glycemic control in T2D: elevated KB are cross-sectionally associated with poorer glycemic control but with better long-term glycemic control. Future studies are needed to extend the idea that the paradoxical role of KB is also present in the context of treatment modalities affecting the metabolism of KB, such as ketogenic diets, and the use of SGLT-2 inhibitors.

## Figures and Tables

**Table 1:** Baseline characteristics of participants in ZODIAC.

	Total (*n* = 271)
Age (years)	65 ± 10
Gender (female, %)	143 (53)
Smoking (yes, %)	45 (17)
BMI (kg/m^2^)	28 ± 5
Systolic blood pressure (mmHg)	152 ± 25
Diastolic blood pressure (mmHg)	84 ± 11
History of cardiovascular disease (%)	100 (37)
eGFR (mL/min/1.73 m^2^)	70 ± 15
HbA1c (%)	7.2 ± 1.2
Total cholesterol (mmol/L)	5.54 ± 1.07
HDL cholesterol (mmol/L)	1.18 ± 0.32
Triglycerides (mmol/L)	2.16 (1.54–3.30)
C-reactive protein (mg/L)	2.70 (1.70–4.40)
Total fasting plasma KB (μmol/L)	182 (147–232)
β-hydroxybutyrate (μmol/L)	120 (93–161)
Acetoacetate (μmol/L)	41 (32–56)
Acetone (μmol/L)	18 (12–25)
T2D therapy	
Diet (%)	29 (11)
Use of OBGLD (%)	152 (56)
Use of insulin (%)	21 (8)
Use of OBGLD and insulin (%)	18 (7)
Unknown (%)	51 (19)
T2D duration (years)	4 (0–51)

Values are means (±standard deviation), medians (interquartile range), or proportions. *BMI:* body mass index; *eGFR:* estimated glomerular filtration rate; *HbA1c:* glycated hemoglobin; *HDL:* high-density lipoprotein; *T2D:* type 2 diabetes; *OBGLD:* oral blood-glucose-lowering drugs.

**Table 2:** Cross-sectional associations between fasting plasma KB and baseline HbA1c levels in linear regression analyses in ZODIAC.

	Total KB *Per Doubling*	*p*	β-OHB *Per Doubling*	*p*	Acetoacetate *Per Doubling*	*p*	Acetone *Per Doubling*	*p*
	B (95% CI)		B (95% CI)		B (95% CI)		B (95% CI)	
Crude	**0.42 (0.17–0.66)**	**0.01**	**0.36 (0.16–0.56)**	**<0.001**	0.02 (−0.13–0.17)	0.82	0.01 (−0.10–0.12)	0.86
Model 1	**0.42 (0.17–0.67)**	**0.01**	**0.36 (0.16–0.56)**	**<0.001**	−0.02 (−0.14–0.09)	0.70	0.01 (−0.14–0.16)	0.89
Model 2	**0.33 (0.10–0.56)**	**0.01**	**0.27 (0.09–0.44)**	**0.01**	−0.04 (−0.16–0.07)	0.46	−0.01 (−0.17–0.15)	0.89
Model 3	**0.39 (0.14–0.64)**	**0.01**	**0.33 (0.13–0.53)**	**<0.001**	−0.02 (−0.14–0.10)	0.77	0.01 (−0.14–0.16)	0.86
Model 4	**0.35 (0.09–0.61)**	**0.01**	**0.30 (0.10–0.51)**	**<0.001**	−0.04 (−0.16–0.08)	0.53	−0.00 (−0.14–0.16)	0.88
Model 5	**0.31 (0.06–0.57)**	**0.02**	**0.28 (0.08–0.47)**	**0.01**	−0.04 (−0.16–0.07)	0.46	−0.02 (−0.19–0.16)	0.86

Model 1: adjusted for age and gender; Model 2: Model 1 + T2D therapy; Model 3: Model 1 + BMI and systolic blood pressure; Model 4: Model 3 + eGFR, smoking, and total cholesterol/HDL ratio; Model 5: Model 4 + T2D duration + C-reactive protein; Coefficients represent the difference in HbA1c (%) levels per doubling of KB. *KB*: ketone bodies; *HbA1c:* glycated hemoglobin; *β-OHB*: β-hydroxybutyrate; *T2D*: type 2 diabetes; *BMI*: body mass index; *eGFR*: estimated glomerular filtration rate; *HDL*: high-density lipoprotein.

**Table 3:** Longitudinal associations between fasting plasma KB and annual HbA1c (%) change over time in linear mixed model analyses in ZODIAC.

	Total KB *Per Doubling*	*p*	β-OHB *Per Doubling*	*p*	Acetoacetate *Per Doubling*	*p*	Acetone *Per Doubling*	*p*
	B(95% CI)		B (95% CI)		B (95% CI)		B (95% CI)	
Crude	**−0.11 (−0.20–−0.02)**	**0.01**	**−0.09 (−0.16–−0.02)**	**0.02**	−0.03 (−0.09–0.04)	0.74	−0.03 (−0.09–0.04)	0.40
Model 1	**−0.11 (−0.20–−0.02)**	**0.01**	**−0.09 (−0.16–−0.02)**	**0.02**	−0.03 (−0.10–0.03)	0.57	−0.03 (−0.10–0.03)	0.35
Model 2	−0.08 (−0.18–0.01)	0.07	−0.07 (−0.14–0.00)	0.06	−0.03 (−0.10–0.03)	0.67	−0.03 (−0.10–0.04)	0.37
Model 3	**−0.11 (−0.20–−0.02)**	**0.01**	**−0.08 (−0.15–−0.01)**	**0.03**	−0.03 (−0.10–0.03)	0.57	−0.03 (−0.09–0.04)	0.37
Model 4	**−0.10 (−0.20–−0.01)**	**0.03**	**−0.08 (−0.16–−0.01)**	**0.03**	−0.03 (−0.10–0.04)	0.76	−0.03 (−0.09–0.04)	0.43
Model 5	**−0.10 (−0.19–−0.00)**	**0.05**	**−0.08 (−0.16–−0.01)**	**0.03**	−0.01 (−0.06–0.04)	0.77	−0.01 (−0.08–0.05)	0.69

Model 1: adjusted for age and gender and baseline HbA1c; Model 2: Model 1 + T2D therapy; Model 3: Model 1 + BMI and systolic blood pressure; Model 4: Model 3 + eGFR, smoking, and total cholesterol/HDL ratio; Model 5: Model 4 + T2D duration + C-reactive protein. Linear mixed model analyses were performed on all individuals with ≥ 2 HbA1c measurements. A *p*-value of <0.05 was considered statistically significant. All models were adjusted for time as the main fixed factor in linear mixed model analyses. The estimates and *p*-values for the interactions of KB with time are depicted. Coefficients represent the yearly change in HbA1c (%) levels per doubling of KB. *KB:* ketone bodies; *HbA1c:* glycated hemoglobin; *β-OHB*: β-hydroxybutyrate; *T2D*: type 2 diabetes; *BMI*: body mass index; *eGFR*: estimated glomerular filtration rate; *HDL*: high-density lipoprotein.

## Supplementary Materials

The following supporting information can be downloaded at: https://www.mdpi.com/article/10.3390/biom12091318/s1, Table S1: Diabetes treatment stratified longitudinal associations between fasting KB and annual HbA1c change in linear mixed models in ZODIAC; Table S2: Longitudinal associations between fasting plasma KB and annual fasting glucose change in linear mixed model analyses in PREVEND; Figure S1: Graphical illustration of the association between KB according to tertiles at baseline and HbA1c at baseline and over time (average).
