# Supplementary material for: The Paradoxical Role of Circulating Ketone Bodies in Glycemic Control of Individuals with Type 2 Diabetes: High Risk, High Reward?
Source: Biomolecules. 2022 Sep 18;12(9):1318. doi: 10.3390/biom12091318 (PMC9496560; doi:10.3390/biom12091318)

Supplemental Table S1. Diabetes treatment stratified longitudinal associations between fasting KB and annual HbA1c change in linear mixed models in ZODIAC

	**Diet alone** (n=29)<br><br>**KB**<br>*per doubling*<br><br>B<br>(95% CI)	***p***	**OBGLD** (n=152)<br><br>**KB**<br>*per doubling*<br><br>B<br>(95% CI)	***p***	**Insulin** (n=21)<br><br>**KB**<br>*per doubling*<br><br>B<br>(95% CI)	***p***	**OBGLD and insulin** (n=18)<br><br>**KB**<br>*per doubling*<br><br>B<br>(95% CI)	
Crude	-0.27 (-0.79 – 0.26)	0.31	**-0.11 (-0.20 - -0.01)**	**0.03**	0.17 (-0.05 – 0.39)	0.15	-0.08 (-0.42 – 0.25)	0.61
Model 1	-0.19 (-0.75 – 0.38)	0.51	**-0.11 (-0.21 - -0.01)**	**0.03**	0.15 (-0.10 – 0.40)	0.24	-0.08 (-0.45 – 0.29)	0.67
Model 2	-0.22 (-0.80 – 0.35)	0.44	**-0.11 (-0.21 - -0.01)**	**0.03**	0.18 (-0.15 – 0.51)	0.28	-0.07 (-0.46 – 0.31)	0.70
Model 3	-0.30 (-0.92 – 0.32)	0.33	**-0.10 (-0.20 - -0.00)**	**0.05**	0.19 (-0.14 – 0.53)	0.25	-0.06 (-0.58 – 0.45)	0.81
Model 4	-0.37 (-1.02 – 0.28)	0.26	**-0.11 (-0.21 - -0.00)**	**0.05**	0.21 (-0.19 – 0.61)	0.30	-0.08 (-0.53 – 0.37)	0.72

Crude
Model 1: adjusted for age and gender and baseline HbA1c
Model 2: Model 1 + BMI and systolic blood pressure
Model 3: Model 2 + eGFR, smoking and total cholesterol/HDL ratio
Model 4: Model 3 + T2D duration + C-reactive protein

Linear mixed model analyses were performed in all individuals with ≥ 2 HbA1c measurements. A p-value of <0.05 was considered statistically significant. All models were adjusted for time as main fixed factor in linear mixed model analyses. The estimates and p-values for the interactions of KB with time are depicted. Coefficients represent the yearly change in HbA1c (%) levels per doubling of KB.

KB: ketone bodies; *HbA1c* glycated haemoglobin; *T2D:* type 2 diabetes; *BMI:* body mass index; *eGFR:* estimated glomerular filtration rate; *HDL:* high density lipoprotein.

Supplemental Table S2. Longitudinal associations between fasting plasma KB and annual fasting glucose change in linear mixed model analyses in PREVEND

	**Total ketone bodies** *Per doubling* B (95% CI)	***p***	**β-OHB** *Per doubling* B (95% CI)	***p***	**Acetoacetate** *Per doubling* B (95% CI)	***p***	**Acetone** *Per doubling* B (95% CI)	***p***
Model 1	**-0.09 (-0.15 – -0.02)**	**0.01**	**-0.08 (-0.15 - -0.02)**	**0.01**	**-0.06 (-0.11 - -0.01)**	**0.02**	-0.04 (-0.09 – 0.01)	0.12
Model 2	**-0.09 (-0.16 – -0.02)**	**0.02**	**-0.08 (-0.15 - -0.02)**	**0.02**	**-0.06 (-0.11 - -0.01)**	**0.03**	-0.03 (-0.09 – 0.02)	0.20
Model 3	**-0.09 (-0.14 – -0.01)**	**0.02**	**-0.08 (-0.13 - -0.01)**	**0.03**	**-0.05 (-0.10 - -0.00)**	**0.04**	-0.02 (-0.07 – 0.03)	0.39
Model 4	**-0.09 (-0.16 – -0.02)**	**0.01**	**-0.08 (-0.15 - -0.02)**	**0.01**	**-0.06 (-0.11 - -0.01)**	**0.02**	-0.04 (-0.09 – 0.01)	0.11
Model 5	**-0.09 (-0.15 – -0.02)**	**0.01**	**-0.08 (-0.14 - -0.01)**	**0.02**	**-0.06 (-0.11 - -0.01)**	**0.02**	-0.04 (-0.09 – 0.01)	0.13

Model 1: adjusted for age and gender and baseline glucose
Model 2: Model 1 + time-updated T2D therapy and baseline plasma insulin concentration
Model 3: Model 2 + time-updated eGFR, systolic blood pressure and use of antihypertensive drugs
Model 4: Model 2 + time-updated BMI, smoking and alcohol use
Model 5: Model 2 + time updated total cholesterol/HDL ratio

Linear mixed model analyses were performed in all individuals with ≥ 2 fasting glucose measurements. A p-value of <0.05 was considered statistically significant. All models were adjusted for time as main fixed factor in linear mixed model analyses. The estimates and p-values for the interactions of KB with time are depicted. Coefficients represent the yearly change in fasting glucose levels (mmol/L) per doubling of KB.

KB: ketone bodies; *HbA1c* glycated haemoglobin; *β-OHB:* β-hydroxybutyrate; *T2D:* type 2 diabetes; *BMI:* body mass index; *eGFR:* estimated glomerular filtration rate; *HDL:* high density lipoprotein.

Supplemental Figure S1. Graphical illustration of the association between KB according to tertiles at baseline and HbA1c at baseline and over time (average).

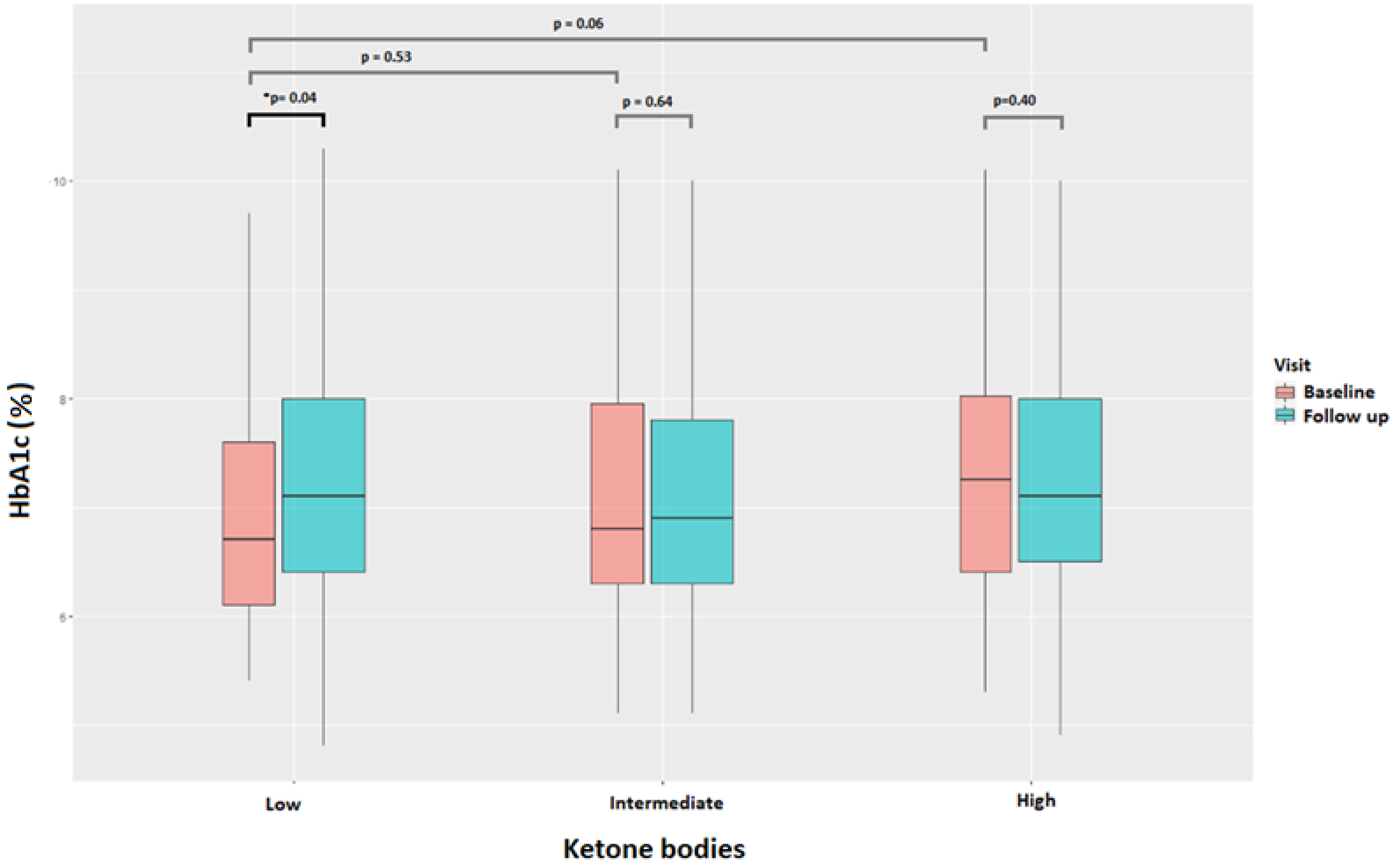

Supplement: Supplementary file 1 [file biomolecules-12-01318-s001.zip › biomolecules-1872856-supplementary.pdf]
